# A Collaborative Psychiatric-Genetics Inpatient Care Delivery Model Improves Access to Clinical Genetic Evaluation, Testing, and Diagnosis for Patients With Neurodevelopmental Disorders

**DOI:** 10.3389/fgene.2022.901458

**Published:** 2022-06-13

**Authors:** Amelle Shillington, Martine Lamy, Kelli C. Dominick, Michael Sorter, Craig A. Erickson, Robert Hopkin

**Affiliations:** ^1^ Cincinnati Children’s Hospital Medical Center, Department of Human Genetics, Cincinnati, OH, United States; ^2^ Cincinnati Children’s Hospital Medical Center Department of Psychiatry, Cincinnati, OH, United States; ^3^ University of Cincinnati College of Medicine Department of Pediatrics, Cincinnati, OH, United States

**Keywords:** autism spectrum disorder, intellectual disability, inpatient child psychiatry, clinical genetics, whole-exome sequencing

## Abstract

Neurodevelopmental disorders including autism spectrum disorder, intellectual disability, and global developmental delay are among the most common indications for referral to clinical genetics evaluation; and clinical genetic testing is indicated for people with neurodevelopmental disorders. There are known barriers to care in accessing clinical genetics evaluation for this patient population. We created a collaborative psychiatric–genetics consultation service and psychiatric–genetics outpatient clinic with the goal to improve care delivery to patients with neurodevelopmental disorders. Two years after the launch of this pilot program, our data demonstrate improved access to genetics evaluation with shorter wait times and fewer patients lost to follow-up. Perhaps most importantly, new genetic diagnoses changed medical care for the majority of patients.

## Introduction

Neurodevelopmental disorders (NDD), including autism spectrum disorder (ASD), intellectual disability (ID), and global developmental delay (GDD), are among the most common indications for referral to clinical genetics evaluation (Mahler et al., 2019). Indeed, identifying a molecular diagnosis for patients presenting with NDD impacts the patient directly, along with their families, with potential benefits including more accurate reproductive counseling, engagement in community supports, connection to disorder-specific support groups, improved prognostic information on comorbidities leading to specific disease monitoring or direct changes in medical management, and the opportunity for participation in gene-specific translational research studies and clinical trials (Besterman et al., 2020). The current American College of Medical Genetics (ACMG) guidelines recommends genetic testing for all patients with NDD (South et al., 2013; Manickam et al., 2021). Even with strong evidence-based recommendations, there are many patients with NDD who do not receive clinical genetics evaluation or diagnostic testing for a variety of reasons, including lack of knowledge about the utility of genetic testing from referring developmental pediatric providers, lack of insurance coverage for genetic testing (Barton et al., 2018), long wait times or lack of access to a genetics provider, parental medical literacy, and lack of understanding about the value of results (Zebolsky et al., 2020), distance to travel for appointments, lower socioeconomic status or underrepresented minority status (Kalb et al., 2012), and patients with severe behaviors who cannot tolerate outpatient exams due to overstimulating waiting rooms without behavioral support staff (Thom et al., 2019) These factors are further complicated by a general lack of access to quality medical care for children with NDD, who are often left without a medical home and have difficulty accessing health care (Cheak-Zamora and Thullen, 2017; Lindly et al., 2019).

Given the challenges of accessing care and establishing a medical home in this population, child and adolescent psychiatrists who work with patients with NDD can find themselves in a unique position in terms of longitudinal care for patients with co-occurring psychiatric disorders. They may be the most consistent point of contact within the healthcare system for those requiring long-term psychiatric medication management. The Neurobehavioral Continuum of Care at Cincinnati Children’s Hospital Medical Center (CCHMC) includes clinicians and staff dedicated to the care of individuals with NDD and co-occurring mental illness and severe behaviors across outpatient and inpatient care settings. Our specialized neurobehavioral inpatient unit (NBU) for patients with NDD, which is part of the Autism & Developmental Disorders Inpatient Research Collaborative (ADDIRC) (Siegel et al., 2015), is part of the larger psychiatric hospital at CCHMC. The NBU accepts admissions for patients with an underlying diagnosis of ID, ASD, or global developmental delay requiring psychiatric stabilization. Most of the patients cared for in the neurobehavioral unit have severe problem behaviors, including aggression toward others or self-injurious behaviors such as head banging. Another common indication for psychiatric admission is induction or discontinuation of behavioral medication, which can require close inpatient monitoring. The neurobehavioral unit has dedicated psychiatrists with an expertise in neurodevelopmental disorders who take care of patients with NDD in the inpatient setting and who also staff a dedicated neurobehavioral psychiatry outpatient clinic where patients are followed for outpatient medication management and care coordination after inpatient discharge. Psychiatrists specializing in NDD in our institution specifically, and broadly across pediatric institutions nationally and internationally are a key source of referrals for genetics evaluations.

With the knowledge that there are many factors that contribute to decreased accessibility to genetics evaluation and multiple drivers of loss to follow-up, our team created an inpatient psychiatric-genetics consultation service as well as an outpatient psychiatric-genetics clinic for previously evaluated or newly referred patients with NDD. Two years after the launch of this pilot program, we performed a retrospective chart review to assess the accessibility of genetics care delivery for patients with NDD. Specifically, we analyzed wait time for genetics appointments for patients referred by neurodevelopmental psychiatrists for evaluation, along with the percentage of no-show rates for genetics follow-up. We compared two cohorts, dividing patients between those referred for genetics evaluation before the implementation of the program (1 March 2019) to those referred after the program’s implementation (through 31 October 2021). Secondary outcomes measured included diagnostic yield in both cohorts, time to diagnosis, and yield of test type (comparing microarray to exome-based testing as a first-line test). Finally, we analyzed changes in care or implementation of syndrome-specific management after a new diagnosis was conferred.

## Methods

A collaborative psychiatric-genetics inpatient consult service and associated outpatient follow-up clinic was established in March of 2019. We created a collaborative inpatient consult service to perform “outpatient visits in the inpatient setting”, completing genetics evaluations during an inpatient psychiatric admission. In addition, we started an outpatient genetics clinic, embedded within the physical psychiatry clinic space, for inpatient follow-up appointments as well as seeing outpatient referrals from neurobehavioral psychiatry. Co-localization of the clinic allowed the genetics team the ability to utilize support tools in place in the neurobehavioral outpatient clinic for patients with behaviors that can make medical visits difficult. For example, the clinic is staffed with specialized support staff (nurses, medical assistants, and behavioral specialists) trained in specific safety procedures for this population as well as regular use of behavioral support tools such as visual schedules. The clinic has a smaller wait area without overwhelming sensory stimuli as well as distraction and other destimulation tools to aid in conducting medical procedures, such as blood draws, more comfortable for this population. Alternatively, families could choose to be seen virtually *via* telemedicine to assist patients having to drive a long way, avoiding long travel times, or to aid patients with behavior difficulties, where traveling by car and/or presenting to waiting rooms was difficult.

At the initial launch of the program, a geneticist and genetics fellow were present at the psychiatric neurobehavioral inpatient unit 2 days per month, offering consultations on admitted inpatients in the morning and conducting telemedicine or in-person outpatient clinic visits in the afternoon to follow up on referrals from neurodevelopmental psychiatrists. Criteria for admission to the neurobehavioral unit are that all patients have a diagnosis of autism and or intellectual disability; thus, by default, all admitted patients had an indication for genetics evaluation. Evaluations were completed during the inpatient stay; however, in some cases, patients were admitted after or discharged before the scheduled inpatient genetics consult day; these patients were referred to the embedded outpatient psychiatric-genetics clinic upon discharge when possible. Patients referred to genetics from the inpatient neurobehavioral unit from 1 March 2019 through 31 October 2021 make up the “post-collaboration cohort” (PostCC). As a comparison, we contrasted our outcomes to a cohort of patients who were cared for by neurodevelopmental psychiatrists and were referred to genetics evaluation prior to the launch of the collaboration, namely, from 1 July 2010 through 1 March 2019. This group makes up the “pre-collaboration cohort” (PreCC). Prior to this collaboration, neurodevelopmental psychiatric inpatients did not have an option for inpatient genetics evaluation; thus, due to systemic limitation, all of these patients were only able to be offered genetics outpatient appointments.

A retrospective chart review was conducted to query the CCHMC Electronic Health Record (EHR) generating a list of patients who had been evaluated by a neurodevelopmental psychiatrist (who by default had a diagnosis of ASD, ID, or GDD) where a referral order was placed to Genetics. We generated a list of 135 patients, 34 had been referred before the initiation of the psychiatric-genetics collaboration (before 1 March 2019), forming the PreCC, and 101 patients had been referred after the launch of the psychiatric-genetics collaboration, forming the PostCC. A review of these charts was completed to document the accessibility to genetics evaluation, genetic test results, and patient outcomes.

Patients in the PostCC were evaluated by a clinical genetics fellow and supervising clinical geneticist, and testing was completed following ACMG guidelines; which included chromosomal microarray, fragile X testing (in selected cases), and if negative, exome or exome-based broad autism/intellectual disability (2000+ gene panel). Patients in the PreCC received an evaluation by a clinical geneticist, and clinical testing was completed based on the recommendation of the evaluating clinician. In patients for whom a new genetic diagnosis was made in the PostCC, a chart review was completed to evaluate if any disease-specific management changes were made, and this was reported.

A two-tailed t-test was performed to compare the mean length of time from referral placement to genetics evaluation between cohorts. A chi-square test was performed to compare the percentage of patients who were lost to follow-up between groups. The overall yield of testing was reported in both groups. Overall length of the diagnostic odyssey was reported in both groups. A comparison of yield was made between microarray and exome-based testing, when both tests were sent concurrently.

## Results

### Demographics

A total of 135 patients were included in this analysis, 34 in the PreCC and 101 in the PostCC. In the PreCC, there were 25 male patients and nine female patients. In the PostCC, there were 71 male patients and 31 female patients. Sex differences were not significant between groups (chi-square = 0.1889. *p* = 0.7). In the PreCC, the mean age of patients at the time of referral was 11.2 years (SD 4.6 years), and in the PostCC, the mean patient age at the time of referral was 13.9 years (SD 4.0 years). The mean age was slightly increased in the PostCC (t = 3.34067, *p* = 0.0005). The PreCC patient population was mostly White Non-Hispanic (88%) as was the PostCC cohort (74%); this difference was not significant (Table 1).

**TABLE 1:** Demographics of study population.

	PreCC (*n* = 34)	PostCC (*n* = 101)	*p* value
Male/female	25/9	71/31	*p* = 0.07
Mean age in years (SD)	11.2 (4.6)	13.9 (4.0)	*p* = 0.0005
Race/ethnicity = White Non-Hispanic (%)	30 (88)	75 (74)	*p* = 0.09

### Main Results

We hypothesized that patients in the PostCC would have a shorter wait time to be evaluated by a geneticist since evaluations were available in the inpatient setting or in an embedded outpatient clinic. In the PreCC, the average number of days passed from when a referral order to genetics was placed to when a patient was evaluated by a geneticist was 460 days. In the PostCC, the average days from when a referral to genetics was placed to when a patient were evaluated by a geneticist was 26 days. This difference was statistically significant (*t* = 5.23, *p*<.00001.) We conclude that the wait time for genetics evaluation was shorter in the PostCC **(** Table 2 **)**

**TABLE 2:** Outcomes of referral placement.

	PreCC (*n* = 34)	PostCC (*n* = 101)	*p* value
Mean number of days from referral placement to genetics evaluation (range)	460 (0–3280)	26 (0–251)	*p* < 0.00001
Patients lost to follow up (%)	11/34 (32%)	1/101 (1%)	*p* < 0.00001

We hypothesized that the loss to follow-up rate would be lower in the PostCC as evaluations were available in the inpatient setting or in an embedded outpatient psychiatric-genetics clinic, with care delivered in tandem with familiar psychiatric providers. In the preCC, the percentage of patients lost to follow up was 32%. In the postCC, the percentage of patients loss to follow-up was 1%. This difference was statistically significant (chi-square = 23.6267, *p*<.00001). We conclude that the lost to follow-up rate is reduced in the PostCC due to improvements in care delivery **(** Table 2 **).**

**FIGURE 1:**
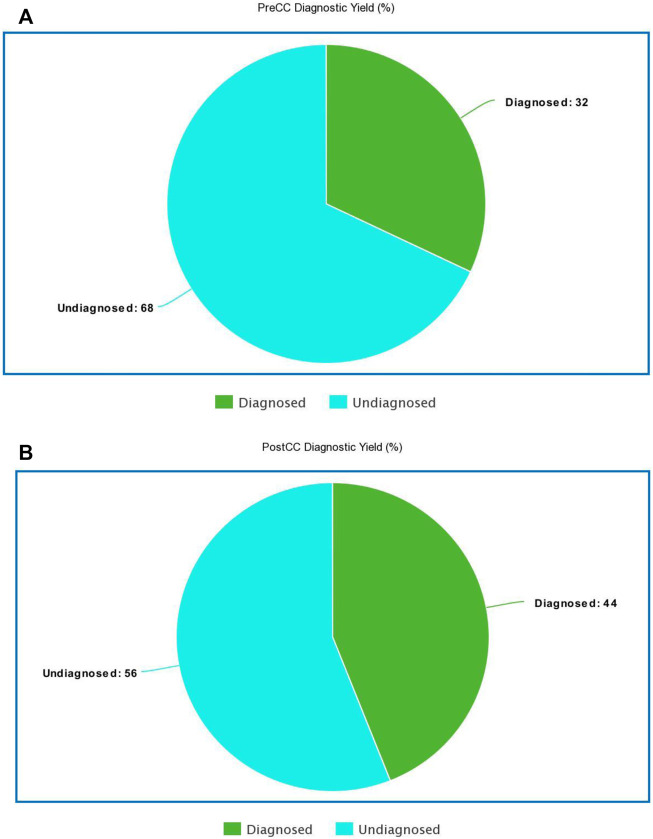
**(A)**: Diagnostic yield in PreCC 1**(B)**: Diagnostic yield of PostCC.

The diagnostic yield (a confirmed molecular diagnosis) for NDD testing previously reported in the literature is near 36% (15%–53%) (Srivastava et al., 2019). The overall diagnostic yield in the PreCC group was 32% (Figure 1A). The overall diagnostic yield in the PostCC group was 44% (Figure 1B). We conclude that the diagnostic yield in this cohort was consistent with that in prior studies.

The diagnostic odyssey for patients with an undiagnosed NDD can be long, often up to 8 years from the initial onset of symptoms to ultimate diagnosis (Michaels-Igbokwe et al., 2021). In this study, across the entire cohort, for patients who were ultimately delivered a new diagnosis, the average time from the initial genetic test (microarray in most cases) to ultimate diagnostic test (by exome sequencing or exome-based panel in most cases) was 678 days (1.85 years) (Supplementary Data Sheet S1). This time-to-diagnosis is consistent with prior literature.

**FIGURE 2:**
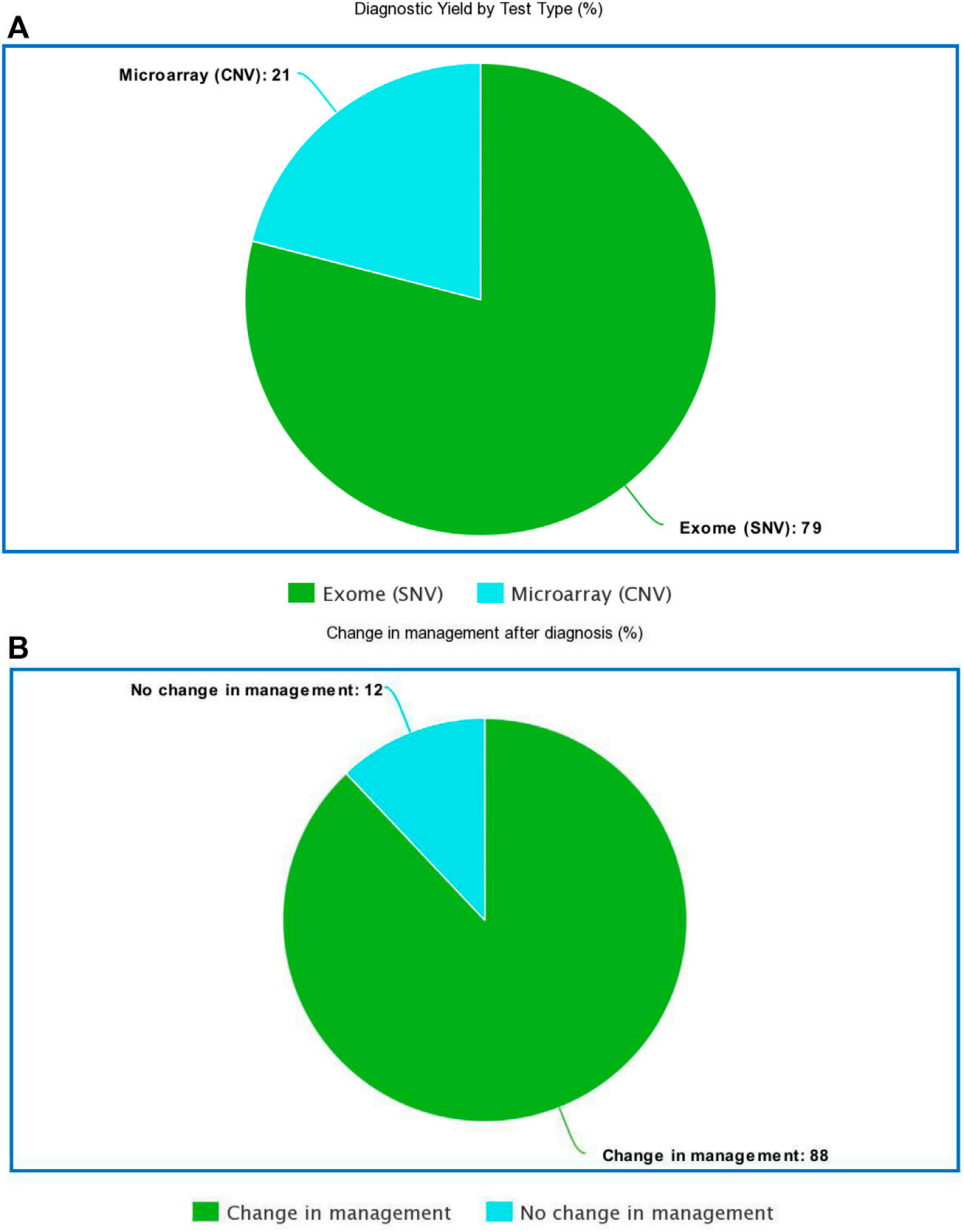
**(A)**: Diagnostic yield by test type **(B)**: Percentage of the diagnosed cohort where disease specific management changes were implemented.

The current ACMG guidelines for genetic testing in NDD recommend a chromosomal microarray as the first-line test for any person with autism spectrum disorder and whole-exome sequencing for anyone with an intellectual disability regardless of autism status (South et al., 2013; Manickam et al., 2021). Most patients in our cohort had comorbid ASD and ID; thus, we elected to send both test modalities. In a small subset of the PostCC, where patients had not received any prior genetic testing, genetic testing was sent concurrently (instead of reflexively) to analyze both single-nucleotide variants (SNVs) (*via* exome-based studies) and copy number variants (CNVs) (*via* microarray or CNV analysis on exome platform). When concurrent testing was pursued and was diagnostic, pathogenic CNVs were diagnosed in 21% (4/19) of cases, whereas pathogenic SNV or small intragenic deletions were detected in 79% (15/19) of diagnosed cases by exome-based sequencing. Exome-based sequencing was nearly four times more likely to identify a diagnosis when both tests were sent concurrently (Figure 2A; Supplementary Data Sheet S1).

Perhaps the most important benefit of genetic testing is implementing disease-specific medical management based on a precise molecular diagnosis. A total of 44 patients evaluated as part of the PostCC received a new diagnosis. Of these 44 patients, disease-specific medical management changes were implemented for 39 patients (88% of cases) (Figure 2B; Supplementary Data Sheet S1). We conclude that the genetic results guided care in newly diagnosed patients.

## Discussion

We created a collaborative psychiatric-genetics consultation service and psychiatric-genetics outpatient clinic with the goal to improve care delivery to patients with NDD. This is the first inpatient genetics consult service to be offered in the inpatient child psychiatry setting. Two years after the launch of this pilot program, the data demonstrate improved access to genetics evaluation, with shorter wait times, and fewer patients lost to follow-up. The diagnostic yield was higher for patients evaluated as part of this collaboration. When testing was sent concurrently, we appreciated higher diagnostic yield from exome-based studies over microarray studies. Perhaps most importantly, new diagnoses changed medical care.

In newly diagnosed patients of the PostCC (*n* = 44), medical management changes were made in 88% of cases, specific to the patient’s newly conferred diagnosis, and are detailed in brief in the Supplementary Data Sheet S1. For example, thirteen patients had medications initiated or changed specifically to their diagnosis (such as initiating mTor inhibitors, changing or adding targeted antiepileptic medication, adding mitochondrial supplements, and switching antipsychotic medication classes). Twelve patients were able to register in disease-specific patient registries, where they were able to connect with families and researchers with expertise in their specific diagnosis. Eight patients had additional family members also received a diagnosis for the same syndrome after they were diagnosed. Five patients had diagnoses that increased cardiomyopathy or congenital heart disease risk, and echocardiogram surveillance was implemented. Three patients were diagnosed with syndromes with significant gastrointestinal comorbidities, and changes were made to their feeding and gastrointestinal symptom management. Three patients were diagnosed with syndromes with endocrinopathy as a feature and were thus initiated on thyroid/calcium/glucose monitoring and screening regimens. Two patients had syndromes with osteoporosis as a comorbidity and were, thus, started on bone density screenings and may be eligible for bisphosphonate infusion in the future. Two patients were diagnosed with syndromes with brain malformations as a comorbidity, and follow-up MRI imaging identified structural abnormalities warranting continued surveillance. Two patients received additional social services after a syndrome-specific diagnosis was made.

Inpatient care delivery of clinical genetics evaluation and testing is a somewhat novel concept, and to our knowledge, we are the first to report on the model of embedding a clinical geneticist into psychiatric acute inpatient care delivery. Prior work in this area included a pilot project trialing a different model, where psychiatric practitioners (fellow physicians) were trained to send genetic testing on inpatients (Bestermen et al., 2020). In their model, genetic testing was initiated during the inpatient setting, and patients were then referred to outpatient clinical genetics providers for result disclosure, variant interpretation, and genetic counseling. In their cohort, a full 40% of patients were lost to follow-up. This finding parallels the loss to follow-up rate we observed in our PreCC and is a limitation of their model and indeed argues for the importance of clinical genetics continuity for this patient population. This is a systemic improvement demonstrated by our model.

Child and adolescent psychiatry providers who take care of patients with NDD are in a unique position to collaborate with genetics providers in the inpatient and outpatient settings. Our data demonstrate that fewer patients are lost to follow-up when clinical genetics care delivery can be provided in a collaborative encounter during an inpatient admission or in an outpatient multidisciplinary clinic. Wait times to visit a clinical genetics provider are reduced when patients can be evaluated during inpatient admissions or during multidisciplinary outpatient clinic appointments. Since many patients with NDD establish a long-term relationship with psychiatric providers for behavioral medication management, offering clinical genetics evaluations in collaboration with these providers allows for patients to be evaluated in a familiar environment, with the benefit of psychiatric support staff to aid in patient encounters with anticipated difficult behaviors, improving the experience for patients and families. This is of particular importance to many of the patients in the cohort who were specifically admitted to inpatient neurobehavioral care due to a pattern of behavior most often marked by severe physical aggression and/or self-injurious behavior, which are features that often inherently lead to reduced access to medical and in particular subspecialist care (McCarthy, 2005). Our model “met patients where they were”, literally and figuratively, to engage this high need and high acuity population with appropriate medical genetics evaluation and treatment. We believe without the approaches utilized, in many cases, patients in this population would have not received the genetic diagnoses made, thus negatively impacting the patients’ present and future overall health and medical care.

Anecdotally, we noticed that physicians and care providers benefited from this partnership also. Two years of collaboration offered an opportunity for learning and sharing in both directions. Geneticists were able to educate psychiatrists on genetic test selection, test interpretation, and functional variant testing. Specific genetic diagnoses allowed psychiatrists to select more targeted medications for many patients. Psychiatrists were able to share knowledge on the diagnosis and treatment of many neurobehavioral disorders with geneticists, which improved the overall phenotyping of patients and recognition of unique syndrome-specific neurobehavioral features of the disease. As CCHMC is an academic hospital, many genetics, neurology, psychiatry, and pediatric physicians-in-training participated as part of our collaborative care team and also were able to learn about genetics diagnostics and psychiatric management for patients with NDD. These future physicians often endorsed a greater understanding of genetic determinants of disease and felt greater empowerment to send genetic testing where appropriate, so it is possible that this program may have downstream effects in future institutions as physicians-in-training move on in their careers. Patients evaluated in this cohort likely benefited from bidirectional knowledge sharing to improve overall care and management.

There are some limitations to our study design. It is difficult to comment on comparisons of test yield and time to diagnosis between the two groups, and indeed this is not the main purpose of this study. Any attempts to do so are difficult to interpret. In the PreCC, patients were evaluated by any available geneticist, and multiple providers exist at our institution and may have different approaches to genetic evaluation and testing. In the PostCC, the same embedded geneticist and genetics fellow evaluated all patients and followed a consistent evaluation and test selection algorithm. The PostCC had the benefit of time as well as patients evaluated before 2016 did not have next-generation sequencing readily available at the time of their evaluation. Patients evaluated before 2021 may not have been offered exome-based sequencing as a preferred or first-line test as this recommendation has only been recently made by academic committees. We would expect a higher test yield with broader studies, and the most recently evaluated patients likely received broader, faster, and more technologically sophisticated testing. In general, the team involved in this project tried to use progressive, innovative problem-solving to reach patients wherever they were. This did not come without challenges. Coordinating trio studies is often arduous in exome-based testing, and procuring trio samples in this cohort was often complicated as it is not common for parents to room in with admitted inpatients. We used the services of genetic counselors, nurses, phlebotomists, and medical assistants to assist in tracking down parents for consent and coordinating blood draws after our official inpatient consult was completed. Dys-synchronous sample collection was less of a problem when parents and patients were present together in the same physical space for outpatient appointments. Initially, we had some delays with rolling out telemedicine options for families and patients; however, the COVID epidemic was serendipitous in timing as the institution quickly invested in hospital-wide telemedicine technology upgrades which proved to be extremely beneficial for this patient population.

Ultimately, this retrospective pilot study inspired institutional changes, with a permanently embedded geneticist now a part of the neurobehavioral psychiatry team to continue to improve care delivery to patients with NDD. Future areas of study include analyzing overall cost savings after a new diagnosis is conferred. We suspect that in some NDD diagnoses, especially those where disease-specific medication changes lead to an improvement in symptoms, hospital stays would be shortened, reducing the cost of medical care. We wonder if those patients who were lost to follow-up in both cohorts have lower socioeconomic status, which complicated outpatient care coordination. If there are patterns of lack of access second to poverty, offering inpatient clinical genetics evaluation becomes of high import from a position of health equity and justice. We also would like to evaluate patient and parent satisfaction. We suspect that improving accessibility to genetics evaluation would increase overall patient satisfaction. Evaluating these and other outcomes is the plan for future work.

## Data Availability

The original contributions presented in the study are included in the article/Supplementary Materials; further inquiries can be directed to the corresponding author.
